# Role of the complement system in the tumor microenvironment

**DOI:** 10.1186/s12935-019-1027-3

**Published:** 2019-11-15

**Authors:** Ronghua Zhang, Qiaofei Liu, Tong Li, Quan Liao, Yupei Zhao

**Affiliations:** Department of General Surgery, Peking Union Medical College Hospital, Peking Union Medical College & Chinese Academy of Medical Sciences, 1# Shuai Fu Yuan, Dong Dan District, Beijing, 100730 China

**Keywords:** Complement system, Tumor microenvironment, Immunoregulation, Immunotherapy

## Abstract

The complement system has traditionally been considered a component of innate immunity against invading pathogens and “nonself” cells. Recent studies have demonstrated the immunoregulatory functions of complement activation in the tumor microenvironment (TME). The TME plays crucial roles in tumorigenesis, progression, metastasis and recurrence. Imbalanced complement activation and the deposition of complement proteins have been demonstrated in many types of tumors. Plasma proteins, receptors, and regulators of complement activation regulate several biological functions of stromal cells in the TME and promote the malignant biological properties of tumors. Interactions between the complement system and cancer cells contribute to the proliferation, epithelial-mesenchymal transition, migration and invasion of tumor cells. In this review, we summarize recent advances related to the function of the complement system in the TME and discuss the therapeutic potential of targeting complement-mediated immunoregulation in cancer immunotherapy.

## Background

Despite the significant advances in the understanding of the immunological basis of cancer, cancer is still an enormous public burden on society [1, 2]. Growing evidence demonstrates that the tumor microenvironment (TME) plays indispensable roles in tumorigenesis, progression, metastasis, recurrence, and drug resistance [3]. The TME is composed of cancer cells, stromal cells and extracellular components [4]. The stromal cells include immune cells and fibroblasts [5]. Tumor-associated macrophages (TAMs), tumor-associated neutrophils (TANs) and myeloid-derived suppressor cells (MDSCs) are populations of immunosuppressive cells that infiltrate in the TME to the greatest extent [6]. Regulatory T cells (Tregs) [7], cancer-associated fibroblasts (CAFs) [8] and dendritic cells (DCs) [9] have also been reported to contribute towards the proliferation and invasion of tumors. Interactions between these cells and cancer cells play crucial roles in tumor malignant biological behavior and therapeutic effects.

The complement system has traditionally been considered a branch of the innate immune response that enhances the effects of antibodies and eliminates cellular debris and foreign intruders [10]. There are three main complement activation pathways: the classical pathway (CP), the lectin pathway (LP), and the alternative pathway (AP). All three pathways merge into a common terminal pathway that includes the activation of complement component 5 (C5) into C5a and C5b. C5b binds to C6 and C7 to form the C5b–C6–C7 complex, which is anchored to cell membranes and interacts with C8 and C9 to form the membrane attack complex (MAC), leading to antibody-mediated complement-dependent cytotoxicity (CDC). After this activation, complement proteins are activated and cleaved, and some of the resultant products are deposited on cell surfaces or released into body fluids to interact with specific receptors. The complement system acts as an efficient immune surveillance system and contributes substantially to homeostasis [10]. However, recent studies provide new perspectives on the immunosuppressive functions of complement components. Studies over the last decade have demonstrated that these complement components could contribute to regulating the function of the TME as a bridge between tumor-promoting and tumor-suppressing immune responses. This review discusses complement system activation in cancer and interactions between the complement and the TME to provide a framework in which to understand the role of the complement system in cancer and discuss the potential of therapies targeting complement activation in the TME.

## Complement activation in the TME

**Fig. 1:**
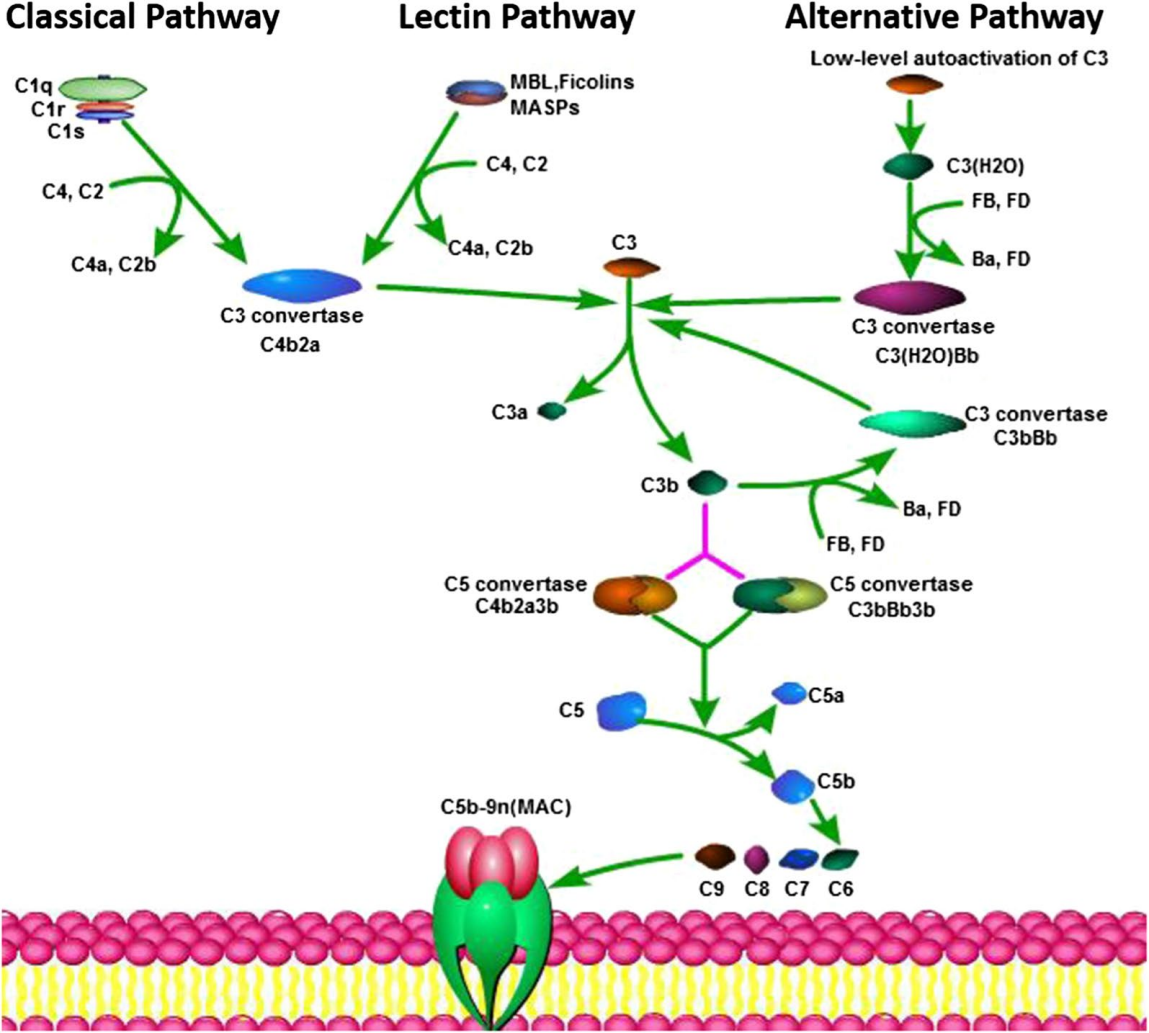
The three pathways of the complement cascade. The complement system has three main complement activation pathways: the classical pathway (CP), the lectin pathway (LP), and the alternative pathway (AP). The CP is triggered by activation of the C1-complex (C1q–C1r–C1s). The LP is homologous to the CP and activated by mannose-binding lectin (MBL), ficolins and mannose-binding protein-associated serine proteases (MASPs). The AP is continuously activated by low-level autoactivation of C3. All the three pathways merge into a common terminal pathway with the activation of C5 into C5a and C5b. C5b binds to C6 and C7 and interacts with C8 and C9 to form the membrane attack complex (MAC)

The complement system is important in regulating humoral immunity and complement proteins are abundant in the immune microenvironment [11]. The complement system is composed of more 50 serum proteins and membrane-bound regulators and receptors that interact with various cells and mediators of the immune system [10, 12]. The complement cascade is summarized in Fig. 1. However, in the presence of malignancy, the balance between the concentrations and proportions of complement components in body fluids was observed to be lost [13, 14]. The expression of complement proteins is increased in malignant tumors, and complement activation in the TME promotes tumorigenesis and progression. The main pathway involved in complement activation in the TME remains unclear. The CP was identified as the main contributor to complement activation in a model of cervical cancer [15]. The LP was found to be significantly increased in colorectal cancer patients compared with healthy persons [16]. The complement system has been reported to be activated in tumor cells and tumor tissues, and these findings are summarized in Table 1. In addition to host cells, tumor cells can produce complement proteins. Increases in C3 and C5a concentrations were observed in the plasma of a mouse model of metastatic breast cancer [17]. C3 cleavage products were extensively deposited along the tumor vasculature in a mouse model of cervical cancer [15]. Tumor cells were shown to secrete C3 in a syngeneic mouse model of ovarian cancer and cancer cell lines, and C3 deposition was found in tumors resected from C3-deficient mice [18]. C4d, a degradation product of complement activation, was found to be elevated in malignant lung tissues, bronchoalveolar lavage fluid, and plasma from lung cancer patients and C4d levels were associated with disease prognosis [19]. C4d fragments were also detected in oral squamous cell carcinomas, and C4d levels in saliva from patients were increased [20]. Deposition of the complement proteins including C1q and C5b-9 was also demonstrated in melanoma and breast, colon, lung, and pancreatic cancer [21–23]. While tumor cells and stromal cells produce aberrant complement proteins, the complement system is pathologically activated in the TME, which reciprocally promotes tumor growth by regulating inflammation; stromal cell immunity; and the proliferation, epithelial-mesenchymal transition (EMT), migration and invasiveness of tumor cells.

**Table 1:** Effects of complement system on the TME and their therapeutic potential for cancer treatment

Complement protein	Malignancy types/models	Functions in the TME	Example dugs	Refs.
C1q	Melanoma (murine models and cell lines), cervical cancer (murine models), breast cancer (cell lines), pancreatic cancer (cell lines), colon cancer (cell lines) and lung cancer (cell lines)	Promote angiogenesis, cell adhesion, proliferation and metastasis independent of complement activation, and inhibit the inflammatory response of macrophages and DCs	No correlational studies	[23, 25, 27, 98]
C3a	Melanoma (murine models, patient samples and cell lines), lung cancer (murine models, patient samples and cell lines), gastric cancer (murine models, patient samples and cell lines), colon cancer (murine models, patient samples and cell lines), breast cancer (patient samples and cell lines), pancreatic cancer (patient samples and cell lines)	Promote tumor growth, metastasis, EMT and angiogenesis; regulate the function of TAMs, MDSCs, DCs and Tregs; and serve as a predictive biomarker for cancer diagnosis and response to cancer treatment	Compstatin (C3-targeted complement inhibitor)	[13, 15, 58, 67, 77, 81]
C3d	Lymphoma (murine models and patient samples)	Serve as a predictive biomarker for response to cancer treatment or the tumor stage	No correlational studies	[121]
C4d	Oral squamous cell carcinoma (patient samples), lung cancer (patient samples)	Serve as a diagnostic and prognostic biomarker for cancer progression	No correlational studies	[19, 20]
C5a	Lung cancer (murine models, patient samples and cell lines), gastric cancer (murine models, patient samples and cell lines), hepatocellular carcinoma (murine models and cell lines), colorectal cancer (murine models and cell lines), breast cancer (murine models and cell lines), ovarian cancer (murine models and cell lines), melanoma (murine models), ovarian cancer (murine models), cervical cancer (murine models)	Promote tumorigenesis, tumor growth, angiogenesis, cell motility and invasiveness and inhibit immune function by inducing MDSCs or decreasing CD8^+^ T cells. Blockade of C5aR significantly reduced MDSCs and the immunomodulators ARG1, CTLA-4, IL-6, IL-10, LAG3, and PDL-1	Eculizumab (C3-targeted complement inhibitor) PMX-53 (C5a/C5aR inhibition)	[17, 39, 44, 66, 73, 109]
C7	Liver cancer (murine models, patient samples and cell lines)	Promote the stemness of liver cancer cells	No correlational studies	[93]
mCRPs	Many types of cancers (murine models, patient samples and cell lines)	Protect cancer cells from MAC-mediated CDC and regulate the response of T cells	Bispecific antibodies	[39, 101, 103, 104]
MBL-MASP	Glioblastoma multiforme (patient samples), colorectal cancer (patient samples), hepatocellular carcinoma (murine models)	Protect against the initiation and progression of glioblastoma and colorectal cancer, while suppressing the growth of hepatocellular carcinoma	No correlational studies	[112–114]
FB	Glioblastoma multiforme (patient samples)	Serum levels of FB were decreased in glioblastoma	No correlational studies	[112]
FH	Liver cancer (murine models, patient samples and cell lines), cutaneous squamous cell cancer (patient samples and cell lines)	Promote the stemness of liver cancer cells and serve as a biomarker for the tumor progression of cutaneous squamous cell cancer	No correlational studies	[93, 94]

## Complement components interact with stromal cells in the TME

### TAMs

TAMs have been reported to contribute to tumor progression [24]. It has been reported that complement proteins could activate and recruit macrophages into tumor tissues. C1q could induce macrophage polarization and suppress macrophage NLRP3 inflammasome activation [25]. Pentraxin 3 (PTX3) could regulate the complement cascade by interacting with C1q and factor H (FH), and PTX3 deficiency resulted in complement activation and the recruitment of tumor-promoting macrophages [26]. Recent studies showed that C1q‐polarized macrophages expressed elevated levels of programmed death-ligand 1 (PD-L1) and PD-L2 and suppressed the proliferation of human allogeneic inflammatory T cells, resulting in Treg proliferation [27]. Tumor cell-derived C3a modulated TAMs by promoting the accumulation and immunosuppressive activity via C3a–C3a receptor (C3aR)-PI3Kγ signaling [28]. C5a was demonstrated to inhibit the production of IL-12 in macrophages [29]. C5a mediated macrophage polarization by activation of the nuclear factor-κB (NF-κB) pathway and C5a receptor (C5aR) expressed on TAMs exhibited a tumor-promoting functional profile in colon cancer liver metastatic lesions [30]. Complement proteins are also expressed by macrophages. C3 produced by macrophages promoted renal fibrosis via IL-17A secretion [31]. C9 played a crucial role in CDC-mediated tumoricidal activity, and hypoxia could downregulate C9 in TAMs, promoting non-small cell lung cancer progression [32]. Macrophages could also regulate the production of complement components. Medler et al. found that urokinase (uPA) -expressing macrophages were critical regulators of C3-independent C5a generation in squamous cell carcinomas and the C5a could foster an immunosuppressive TME during carcinogenesis by activating C5aR1^+^ macrophages [33]. The cytokine production such as IL-17A, IL-17F, and IL-23 of TAMs could also be modulated by the complement proteins via the PI3K/Akt signaling cascade [34], suggesting dual regulation by TAMs and the complement system.

### TANs

Neutrophils are the first responders among inflammatory cells during the acute phase of damage and infection. Recent studies showed that neutrophils in the TME, also called TANs, are associated with cancer progression [35]. Activation of the complement system could also induce the accumulation and differentiation of TANs within tumors. Allendorf et al. found that C5a could facilitate neutrophil recruitment by stimulating epithelial and endothelial cells to release leukotriene B4 (LTB4) [36], and Dick et al. found that C5aR induced neutrophil dysfunction [37]. C5a generated upon complement activation increased neutrophil recruitment by promoting IL-1 production [38]. C5aR deficiency could inhibit the tumor metastasis of colon cancer by reducing neutrophil infiltration in metastatic foci in the liver [39]. C5a has also been shown to stimulate the production of functionally active tissue factor (TF) in peripheral blood neutrophils, which resulted in enhanced tumor growth and metastasis formation [40]. It was also shown that coagulation induced by C3aR-dependent neutrophil extracellular traps (NETs) could cause the accumulation of neutrophils with a pro-tumorigenic N2 phenotype during intestinal tumorigenesis [41]. In a model of intestinal ischemia–reperfusion injury, C3aR constrained neutrophil mobilization [42]. Therefore, imbalanced complement activation in the TME could reduce neutrophil proinflammatory function, resulting in a pro-tumorigenic TME, via activation and polarization of TANs to N2-type TANs.

### MDSCs

MDSCs were reported to protect cancer cells from the immune system and modulate immune cell polarization in the TME [43]. In a model of HPV-induced cancer, C5a acted as a potent chemoattractant of MDSCs to primary tumors [15]. Cancer cell-derived C5a could create a favorable TME for lung cancer progression and blockade of C5aR significantly reduced MDSCs [44]. When MDSCs enter the TME, they can suppress the function of T cells via C5a/C5aR pathways. Studies of mouse models of melanoma and breast and lung cancer showed that C5aR-mediated pathways are linked to the differentiation of MDSCs and activation of these pathways is associated with the production of immunomodulators such as arginase-1 (Arg-1), IL-10, TGF-β1, cytotoxic T lymphocyte antigen 4 (CTLA4) and PDL-1 [17, 44, 45]. Then, immunomodulators induced by C5a/C5aR facilitated cancer metastasis by the suppressing T cell responses. It was also shown that C5a/C5aR signaling regulated synthesis of reactive oxygen in MDSCs and inhibited the antigen-specific responses of CD8^+^ T cells [46]. These studies demonstrated that tumor cell-derived C5a induced the recruitment and differentiation of MDSCs into the TME and that MDSCs exerted immunosuppressive effects by altering T cell responses, resulting in tumor progression. MDSCs, the cornerstone of the immunosuppressive shield in the TME, can promote the formation of Tregs and TAMs to protect tumor cells from the immune system and immunotherapy [43]. Therefore, the link between MDSCs and complement activation is important for the formation of an immunosuppressive TME.

### Tregs

Tregs are the immunosuppressive subsets of CD4^+^ T cells that can suppress antitumor immune responses in multiple ways [47]. Tregs are recruited by tumor cells and other stromal cells into the TME, where they play immunosuppressive roles in tumor progression. Importantly, MDSCs play critical roles in the generation of Tregs in the TME, and the connection between complement activation and Tregs is therefore arguably important. Complement activation via a C3aR pathway altered CD4^+^ T lymphocytes and mediated cancer progression in mouse models of lung cancer [48]. The protumor effect of the C5a–C5aR signaling axis was also demonstrated in mouse breast cancer models. In a model of metastatic breast cancer, C5aR inhibited the recruitment and functions of CD4^+^ and CD8^+^ effector T cells in the lung and liver, and the numbers of Tregs in the lungs of C5aR-deficient mice was reduced [17]. C5aR in MDSCs was also found to contribute to the polarization of CD4^+^ T cells in the lungs to Th2 type T cells. The reduced generation of Tregs in the absence of C5aR signaling was also associated with the reduced production of TGF-β1 [49]. C5a inhibition in combination with chemotherapy fostered TME reprogramming, resulting in CD8^+^ T cell-dependent antitumor immune responses [33]. Therefore, the combination of complement inhibition with other therapeutic interventions is more likely to substantially benefit cancer patients.

### DCs

DCs, major players in the control of cancer by adaptive immunity, are traditionally divided into plasmacytoid DCs (pDCs) and conventional DCs (cDCs) [50]. Some studies have demonstrated that DCs could be pro- or anti-tumorigenic depending on the status of the TME [51]. In a p53/KRAS-inducible mouse model of ovarian cancer progression, the depletion of DCs early in the disease course accelerated tumor expansion, but DC depletion at advanced stages of disease significantly delayed aggressive malignant progression. Phenotypically divergent DCs both drove immunosurveillance and accelerated malignant growth [52]. Activation of the complement system exerted both protective and immunosuppressive functions in immune-inflammatory responses against injury and cancer. The complement inhibitors C4b-binding protein (C4BP) and FH were reported to play a critical role in modulating adaptive immune responses by generating an anti-inflammatory state in monocyte-derived DCs [53]. Generation of this type of DCs was accompanied by impaired CD4^+^ T cell proliferation and inhibited IFN-γ secretion. In a plant virus-infected model, C3 depletion in mice increased IFN-α production and the immunotherapeutic properties of immune cells [54]. In an HIV-infected model, complement activation could inhibit the pro-inflammatory functions of DCs [55], and this opsonization function of complement on DCs was also demonstrated in a herpes simplex virus 2 (HSV-2)-infected model [56]. In addition, the production of complement components by DCs was shown to affect their ability to regulate T cell responses. FH produced by DCs could inhibit CD4^+^ T cell proliferation [57], and C1q-polarized DCs expressed higher levels of surface PD-L2 and exhibited decreased autologous Th17 and Th1 cell proliferation [27]. C3 production in DCs was increased by lithium via GSK-3 inhibition and regulated interactions between microglia and neurons [58]. C3a and C5a were also shown to be central mediators of radiotherapy-induced, tumor-specific immunity and clinical response [59]. Therefore, it is tempting to speculate that the antigen-presenting function of DCs is limited in the presence of complement activation and that these DCs carry out their anti-inflammatory function by inhibiting T cell anti-tumor responses.

### CAFs

CAFs are abundant and important stromal cells in the TME that contribute to malignant initiation and progression [60]. Different CAF populations that secrete distinct cytokine profiles have been identified in a variety of cancers, suggesting that different fibroblast subsets may carry out different functions in cancer progression [61]. CAFs were also reported to neutralize the anti-tumor effect of immunotherapy by inducing the infiltration of MDSCs in tumors [62]. Combined depletion of CAFs in the TME and anti-CTLA4 immunotherapy improved therapeutic effects and prolonged animal survival [63]. Recent studies have shown that CD10 and GPR77 expression could specifically define a CAF subset correlated with chemoresistance and poor survival in breast and lung cancer patients [64]. GPR77 has been regarded as a C5aR in the complement signaling pathway [65]. CD10^+^GPR77^+^ CAFs could induce cancer stem cell (CSC) enrichment and chemoresistance by secreting IL-6 and IL-8, and CAFs also produced complement for self-sustained GPR77 signaling. Combined chemotherapy and anti-GPR77 neutralizing antibody could inhibit tumorigenesis and enhance chemotherapeutic effects. Thus, CD10^+^GPR77^+^CAF infiltration may serve as a promising clinical biomarker to predict chemotherapy response.

## Effects of the complement system on tumor progression

### Proliferation

Imbalanced tumor cell proliferation and apoptosis is a distinct characteristic of carcinogenesis. There is some evidence that complement activation in the TME directly or indirectly enhanced tumor cell proliferation. Min et al. [18] showed that tumor-derived C3a and C5a played distinctly important roles in promoting tumor proliferation and that C3 or C5 silencing reduced tumor growth in vivo. In this study, C3aR and C5aR agonists increased the proliferation of ovarian cancer cells, while C3aR and C5aR antagonists decreased the proliferation of these cells. There was also no significant difference in complement effects on tumor proliferation in CD8^−/−^ and WT mouse models, which indicated that complement effects on tumor proliferation were independent of T cells. Other studies have demonstrated the complement-induced promotion of cancer proliferation, in which complement components exerted an indirect effect via regulating the immune response of immune cells [44, 66, 67]. Corrales and colleagues found that C5a promoted cell proliferation and tumor growth in the Lewis lung cancer model by creating an immunosuppressive microenvironment. The blockade of C5aR significantly reduced MDSCs and immunomodulators, inhibiting tumor growth [44]. In ovarian tumor-bearing mice, C5a-expressing tumor cells in an overall immunosuppressive state exhibited accelerated growth, and significantly lower percentages of infiltrating CD4^+^ and CD8^+^ T cells were observed in the spleen and tumors [66]. C3a–C3aR signaling was also reported to participate in promoting tumor proliferation. Jamileh et al. showed that C3a–C3aR signaling contributed to melanoma growth by inhibiting neutrophil and CD4^+^ T cell responses [67]. In conclusion, cancer cells have the capacity to generate C3a and C5a, which can promote cancer cell proliferation and create an immunosuppressive TME for cancer progression. In addition, Agostinis et al. showed that high levels of C1q expression in malignant pleural mesothelioma enhanced tumor adhesion and proliferation via enhanced ERK1/2, SAPK/JNK, and p38 phosphorylation [68]. These results provide a new understanding of the role of the complement system in cancer proliferation and have significant implications for innovative therapeutic and biomarker strategies for some cancers.

### EMT

EMT, the transition between epithelial and mesenchymal phenotypes, contributes to embryonic development and carcinoma progression [69, 70]. The complement system participates in mediating EMT in multiple tumor models. C3a secreted by ovarian cancer cells could induce a reduction in E-cadherin expression and promote EMT in cancer cells, which was regulated by the transcription factor TWIST1 [71]. The contribution of C3 to EMT in renal fibrosis and injury has also been investigated [72]. In addition, activation of C5aR by C5a in hepatocellular carcinoma could induce EMT by downregulating E-cadherin and claudin-1 expression, and upregulating Snail expression via activation of the ERK1/2 pathway [73]. Furthermore, C5aR blockade could impair the migration of lung cancer cells and up-regulate E-cadherin protein expression [74]. TGF-β is a major driving force of EMT. TGF-β-induced EMT in lung cancer cells was reported to confer resistance to CDC by upregulation in the CD59 expression on the surface of cancer cells [75]. In this study, CD59 inhibition was demonstrated to enhance the efficacy of antibody-mediated CDC and inhibit metastasis in lung cancer. Stromal cells stimulated by complement components in the TME, such as TAMs and MDSCs, could also produce TGF-β1 and promote EMT. Therapeutic strategies that inhibit complement pathways may be a promising method to inhibit EMT and limit distant metastasis.

### Metastasis

Tumor metastasis, a process by which tumor cells spread from a primary site to distant organs, is very common in the late stages of cancer [76]. Recent studies have shown that imbalanced complement activation and inflammation also triggered metastatic pathways in various cancer models by enhancing the motility of cancer cells, regulating the status of the TME, degrading the extracellular matrix (ECM) and disrupting tissue barriers. Activation of C3a-C3aR signaling was shown to play an important role in guiding collective cell migration [77]. Activation of the complement cascade in leukemia/lymphoma patients enhanced the motility of malignant cells by downregulating the expression of HO-1 [78]. C5a–C5aR signaling facilitated breast cancer metastasis by promoting Treg generation and suppressing T cell responses in the lungs [17]. C5a generated by colon cancer cells contributed to tumor metastasis by increasing the expression of monocyte chemoattractant protein-1 (MCP-1), IL-10, Arg-1 and TGF-β1 [39]. C3 and C4 could bind to collagen and elastin in the vascular wall, leading to increased vascular stiffness [79]. The C5a–C5aR interaction could induce the expression of MMP-1 and MMP-9, which were important for degradation of the ECM, by the activation of NF-κB and AP-1 [80]. C3a–C3aR signaling in the choroid plexus epithelium has been further demonstrated to disrupt the blood-cerebrospinal fluid barrier and promote cancer cell leptomeningeal metastasis [81]. In addition, complement-regulated TAMs and MDSCs could also promote cancer metastasis by modulating the TME, as mentioned above. Surprisingly, some complement components were shown to carry out functions to promote cancer metastasis independent of complement activation. In a mouse model of melanoma, more lung metastases were observed in WT mice than in C1q-deficient mice, suggesting that C1q promoted cancer metastasis [23]. Further investigations to define the underlying mechanisms of these C1q-mediated effects should be carried out.

### Angiogenesis

Tumor angiogenesis is a key step in cancer progression. Tumor cells secrete pro-angiogenic factors to promote the development of abnormal vascular networks and normalization of the tumor vasculature has emerged as a new strategy for therapeutic cancer management [82]. The role of the complement system in angiogenesis is controversial. There is some evidence of the proangiogenic effects of complement components. In a transgenic mouse model of ovarian cancer, complement inhibition by C3 or C5aR knockout inhibited tumor growth by altering endothelial cell function and vascular endothelial growth factor (VEGF) expression [83]. VEGF was shown to carry out a critical function in vascularization under physiological and pathological conditions [84]. Factors promoting angiogenesis could also be secreted from TAMs or MDSCs, which could be recruited by C3a or C5a to the premetastatic niche [85, 86]. Therefore, the complement system may indirectly contribute to angiogenesis by regulating stromal cells in the TME. However, some findings have provided opposite evidence that C3a and C5a might exert an antiangiogenic effect on the course of pathological postnatal neovascularization in the retina [87]. In addition, evidence has shown that the genetic deletion of C3 or C5aR and pharmacological blockade of C5aR impaired the ability of T cells to overcome the endothelial barrier, infiltrate tumors, and control tumor progression in vivo [88]. In genetic chimera mouse models, local complement activation was demonstrated to disrupt the tumor endothelial barrier, which promoted the successful homing of T cells. Nevertheless, complement activation of C3a or C5a did not contribute to tumor angiogenesis in murine models of lung and cervical cancer or epithelial carcinogenesis [15, 44, 89]. During would healing, C1q was shown to be very effective in inducing an angiogenic phenotype in cultured endothelial cells in vitro and forming new vessels in mice or rats [90]. The effects of complement on tumor angiogenesis are complicated, and the roles of complement components in tumor angiogenesis require further investigation.

### Stemness

CSCs are a subset of cells that possess the capacity to self-renew, differentiate, and give rise to cancer recurrence [91, 92]. There is some evidence supporting the possible effects of the complement system on stemness. A recent report showed that the complement proteins C7 and FH were upregulated in liver tumor-initiating cells, and these proteins were needed to control the stemness of liver cancer cells via LSF-1 [93]. CSF was also reported to be upregulated in cutaneous squamous cell carcinoma and knockdown of CFH expression inhibited the proliferation and migration of these cancer cells by inhibiting basal ERK1/2 activation [94]. In contrast, the role of C7 is not well understood. The complement regulator CD59 was also reported to be upregulated by SOX2 to protect epithelial CSCs to evade complement surveillance [95]. In addition, Lee and colleagues found that the mobilization of hematopoietic stem cells in C5-deficient mice was impaired and that C5a-mediated pro-mobilization effects were mediated by the stimulation of granulocytes rather than hematopoietic stem cells [96]. Another study showed that the contribution of C5a to hematopoietic stem cell mobilization was mediated by C5aR, and C5aR antagonists diminished the pro-mobilization effects of C5a [97]. There are also some studies on the regulation of the stemness-associated signaling pathway by complement activation. Naito and colleagues found that the complement C1q could bind to Frizzled receptors and activate canonical Wnt signaling to promote the aging-associated impairment of muscle regeneration [98] and that Wnt signaling pathways could regulate cancer stemness in various manners [99, 100].

### Immunosuppression

CDC is an effector function mediated by antibody-dependent cytotoxicity resulting in formation of the MAC. Products of complement activation have been shown to participate in immunosuppression by upregulating the expression of molecules such as PDL-1, IL-10, Arg-1, and TGF-β1 and regulating immune cell differentiation. In addition, the expression of membrane-bound complement regulatory proteins (mCRPs), including CD46, CD55 and CD59, is upregulated in many types of cancer cells, which can dwarf antitumor therapeutic efficacy. It has been reported that CD46, CD55 and CD59 could protect cancer cells from MAC-mediated CDC [101–103]. In addition, mCRPs have been shown to regulate T cell responses. John and colleagues showed that CD46 participated in switching T cells towards a regulatory phenotype by attenuating IL-2 production via the transcriptional regulator ICER/CREM and upregulating IL-10 expression via the serine-threonine kinase SPAK [104]. CD59 was demonstrated to downmodulate CD4^+^ T cell activity and CD59 blockade enhanced antigen-specific CD4^+^ T cell responses [105]. Neutralization or blockade of mCRPs in cancer cells could increase the efficacy of antibody-based immunotherapy [106, 107]. Strategies involving regulating the function of mCRPs and the status of immune cells may provide new insights into cancer immunotherapy. However, considering the wide distribution of mCRPs on somatic cells, more studies are needed to further validate the specificity of these treatments.

## Therapeutic potential of targeting complement activation in the TME

**Fig. 2:**
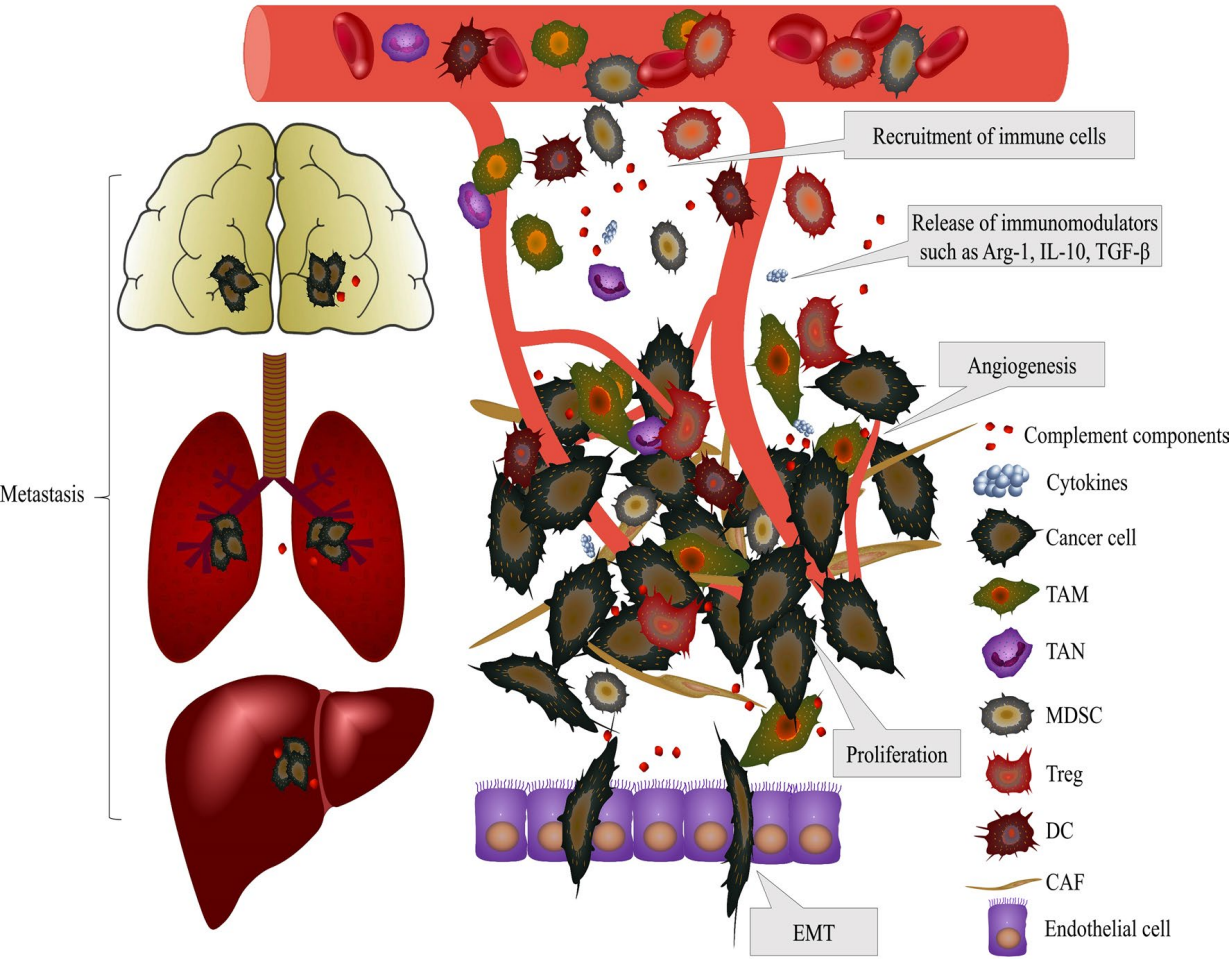
Effects of complement activation on the TME. Activation of the complement system inside tumors releases complement components, such as C1q, C3a, and C5a, into the TME and promotes tumor carcinogenesis. These complement components induce the recruitment of immune cells, including TAMs, TANs, MDSCs, Tregs and DCs, into the tumor. These cells differentiate into tumor-promoting phenotypes and contribute to tumor progression at the following different levels: cell proliferation, angiogenesis, EMT, invasion and metastasis and the inhibition of antitumor immunity. Immunosuppressive cytokines, such as Arg-1, IL-10 and TGF-β, are also released. Imbalanced complement activation and inflammation also promote cancer metastasis into the brain, lung and liver by degrading the ECM and disrupting tissue barriers

The recent clinical success of immune checkpoint blockade suggests that treatment targeting the immune system is the most promising approach to eliminate cancer cells [108]. The multiple roles of the complement system in cancer progression, which is summarized in Fig. 2, have unveiled novel opportunities for the improved management of cancer patients. There is some evidence indicating the therapeutic possibility of complement components as biomarkers or targets for immunotherapies. Serum C3a and C5a have been found to be elevated in patients with lung, colorectal and gastric cancers compared to healthy individuals [109–111]. C4d deposition in tumors was also suggested to serve as a biomarker for the early diagnosis and prognosis of lung cancer [19]. Serum levels of factor B (FB) were decreased and serum levels of mannose-binding lectin (MBL) were elevated in patients with glial tumor, suggesting that low levels of MBL might protect against the initiation and progression of glioblastoma multiforme [112]; furthermore, high serum levels of mannan-binding lectin-associated serine protease 2 (MASP-2) predicted recurrence and poor survival in colorectal cancer patients [113]. However, MBL was reported to suppress tumor growth by regulating hepatic stellate cell activation in a mouse model of hepatocellular carcinoma [114]. Some studies have suggested that the levels of complement proteins served as predictive biomarkers for the response to cancer treatment. Zhang et al. have suggested that C3a was at higher level in samples after neoadjuvant chemotherapy than in that before treatment indicating that C3a might serve as a biomarker to predict the sensitivity of breast cancer to neoadjuvant chemotherapy [115]. Maher et al. demonstrated that serum levels of C4a and C3a might act as predictive biomarkers of the response of esophageal cancer patients to chemoradiation [116]. They found that serum C4a and C3a levels were significantly higher in poor responders versus good responders and these proteins could predict response to neoadjuvant chemoradiotherapy with a sensitivity and specificity of 78.6% and 83.3% in esophageal cancer. Surace et al. found that radiotherapy induced intratumoral complement activation in melanoma and colon carcinoma and C3aR or C5aR blockade before applying radiotherapy could affect antitumor effect of radiotherapy by disturbing DC and CD8^+^ T cell activation. Various studies have suggested the potential application of complement components as cancer biomarkers for the diagnosis, prognosis or response to cancer treatment. However, evidence of their specificity and sensitivity remains insufficient, and the exact mechanisms are still unclear.

PD-1/PD-L1 checkpoint blockades have been shown to be remarkably clinically efficient strategies for various malignancies [117]. C5a blockade was demonstrated to work synergistically with anti-PD-1 inhibition in melanoma and colon and lung cancer growth associated with the activation of CD8^+^ T cells and inhibition of MDSCs [118, 119]. In addition, the targeting of mCRPs, such as CD46 and CD59, for cancer immunotherapy has recently been explored. An antibody–drug conjugate targeting CD46 was shown to eliminate myeloma growth [120], and bispecific antibodies targeting tumor-associated antigens and CD59 increased the efficacy of immunotherapy in a lymphoma mouse model [106]. However, these studies were limited to animal experiments, and differences between the mouse and human complement systems should be considered. There are many challenges and constraints that could hinder development and application in this area. A more detailed understanding of the complex network established between the complement system and cancer is essential to bridge the gap between promising preclinical trials and effective clinical treatments.

## Conclusions and perspective

Recent studies have shown the complex and multifaceted role of complement proteins in immune regulation and cancer. Complement components have been shown to contribute to regulating the functions of the TME and exert immunoregulatory effects under certain conditions. Although we have gained knowledge about the role of the complement system in cancer, molecules that activate the complement cascade in cancer cells are essentially unknown. Due to the high heterogeneity of human cancer, different complement activation pathways and mechanisms may be involved, and different strategies to treat different tumor types could be combined with traditional chemotherapies or immunotherapies. A better understanding of the mechanistic interaction between the complement system and TME will provide a new breakthrough in cancer immunotherapy. In conclusion, targeting complement reagents might be a promising challenge in cancer immunotherapy, and we hope that more efficient therapeutic strategies are developed to improve the efficacy of complement-related anticancer therapies.

## Abbreviations

AP: alternative pathway
Arg-1: arginase-1
C4BP: C4b-binding protein
C5aR: C5a receptor
CAFs: cancer-associated fibroblasts
CDC: complement-dependent cytotoxicity
cDCs: conventional DCs
CP: classical pathway
CSC: cancer stem cell
CTLA4: cytotoxic T lymphocyte antigen 4
DCs: dendritic cells
ECM: extracellular matrix
EMT: epithelial–mesenchymal transition
FB: factor B
FH: factor H
HSV-2: herpes simplex virus 2
LP: lectin pathway
LTB4: leukotriene B4
MAC: membrane attack complex
MASP: mannan-binding lectin-associated serine protease
MBL: mannan-binding lectin
MCP-1: monocyte chemoattractant protein-1
mCRPs: membrane-bound complement regulatory proteins
MDSCs: myeloid-derived suppressor cells
NETs: neutrophil extracellular traps
NF-κB: nuclear factor-κB
pDCs: plasmacytoid DCs
PD-L1: programmed death-ligand 1
PTX3: pentraxin 3
TAMs: tumor-associated macrophages
TANs: tumor-associated neutrophils
TF: tissue factor
TME: tumor microenvironment
Tregs: regulatory T cells
VEGF: vascular endothelial growth factor
